# Clinical and functional outcomes of the PCCP study: a multi-center prospective study in Italy

**DOI:** 10.1007/s11751-013-0159-6

**Published:** 2013-03-31

**Authors:** G. Antonini, R. Giancola, D. Berruti, E. Blanchietti, P. Pecchia, V. Francione, P. Greco, T. C. Russo, L. Pietrogrande

**Affiliations:** 1 Orthopaedic and Trauma Department, Azienda Ospedaliera San Carlo Borromeo, Via Pio II 3, 20153 Milan, Italy; 2 Orthopaedic and Trauma Department, Azienda Ospedaliera San Gerardo, Via Pergolesi 33, 20900 Monza, Italy; 3 Orthopaedic and Trauma Department, Ospedale Civico, Via Po 11, 10034 Chivasso, Italy; 4 Orthopaedic and Trauma Department, Ospedale Santa Maria della Misericordia, Via Comandino 70, 61029 Urbino, Italy; 5 Orthopaedic and Trauma Department, Ospedale G. Mazzini, Piazza Italia, 64100 Teramo, Italy; 6 Present Address: Villa Anna, San Benedetto del Tronto, Via Toscana, 63074 San Benedetto del Tronto, Italy; 7 Orthopaedic and Trauma Department, Azienda Ospedaliera Sant’Anna e San Sebastiano, Via F. Palasciano, 81100 Caserta, Italy; 8 Orthopaedic and Trauma Department, Ospedale Maggiore, Via della Resistenza Partigiana, 1, 97015 Modica, Italy; 9 Operative Unit of Orthopaedics and Traumatology, Dipartimento di Scienze della Salute, Azienda Ospedaliera San Paolo Polo, Università degli Studi di Milano, Via A. Di Rudiní 8, 20142 Milan, Italy

**Keywords:** Functional recovery, Percutaneous compression plate, Intertrochanteric femoral fracture, Reduced blood loss, Reduced transfusions, Femoral fixation

## Abstract

The standard surgical management of hip fractures is associated with tissue trauma and bleeding which are added to the fracture injury. The percutaneous compression plate (PCCP) is a minimally invasive device that has been demonstrated in previous studies to reduce postoperative complications and blood loss. This prospective, multi-center, observational study assessed clinical and functional outcomes with PCCP as treatment for trochanteric fractures. Patients with a stable or unstable proximal femoral fracture of type AO 31.A1 or 31.A2 were enrolled in eight hospitals in Italy. The primary outcome of interest was the recovery of the pre-fracture functional status at 1-year follow-up; secondary outcomes of interest included blood transfusions, surgical time, complications, and mortality. A total of 273 patients were enrolled. The ASA score was 3 or 4 in 72.5 % of patients. The mean surgical time was 44.1 min; the mean post-surgery blood transfusions was 0.9 units. At 1 year, 48 patients (17.6 %) died, 28 (10.2 %) were lost to follow-up, 4 patients (1.5 %) were excluded, hence 193 patients (70.3 %) were available for final evaluation. At the 1-year follow-up visit, 51.9 % of patients recovered or improved their pre-fracture modified Harris Hip Score, 49.1 % of patients improved or maintained their walking abilities, and 66.6 % of patients residing at home pre-surgery maintained their domicile. The overall mortality rate was 17.6 %. Major complications included two fracture collapses, one excessive sliding of the cephalic screw leading to a partial fracture collapse and one back-out of the diaphyseal screw. This study demonstrates that treatment of trochanteric fractures with PCCP gives good outcomes and significant advantages such as low blood loss, short surgical time, low risk of complications, and good functional recovery in the majority of the patients.

## Introduction

Hip fractures are common worldwide [1], with a higher incidence expected in the next few decades due to longer life expectancy and an increase in the geriatric population [2]. Hip fractures usually involve the femoral neck or the trochanteric region and are associated with increased morbidity and mortality, especially in the elderly population [3]. The primary goal of treatment is to obtain an early restoration of the patient’s pre-fracture status, which is best accomplished by early mobilization following surgery [4, 5]. The painful and disabling nature of the hip fractures needs surgical management, even in those patients with little potential for functional recovery [6]. Surgical treatment aims at restoring the ambulatory skills of the patient, and the post-surgical outcomes have been related to stabilization and accuracy of fracture reduction [7]. Beringer et al. [8] reported that the recovery of pre-injury mobility in hip fractures was influenced by the patient’s American Society of Anesthesiologists (ASA) score, age, and domicile status. The functional outcomes following hip fractures in elderly patients have been previously evaluated considering their 1-year mortality rate, recovery of pre-fracture ambulatory status, need for nursing home care, and the ability to live independently [9]. It has been shown that maximum functional recovery occurs within 6 months post-fracture [10], but continues for at least 1 year in some physical and instrumental functions, and beyond 1 year in the patient’s daily physical activities [11].

The surgical treatment of trochanteric hip fractures includes a wide variety of implants and fixation strategies [12]; however, most of these devices and techniques are invasive, are associated with high tissue trauma and high incidence of bleeding, and may worsen the existing comorbidities in elderly patients [13] with the risk of delay in the recovery from the fracture. Therefore, methods of osteosynthesis that reduce the amount of tissue injury, bleeding, and complications, and ensuring a mechanical stability allows early mobilization and rapid return to pre-injury levels of independence are preferred [14]. In an attempt to reduce mortality and accelerate rehabilitation, Gotfried [15, 16] developed the percutaneous compression plate (PCCP), which is a device for minimal-approach osteosynthesis of trochanteric hip fractures [17]. PCCP provides a complete fracture stabilization and fixation, against bending as well as rotational forces, thanks to the presence of two cervical screws, the small screw diameter, that spares bone, and the strength of the plate, that permit full-weight bearing immediately post-surgery [16].

Percutaneous compression plate consists of a plate with three diaphyseal screws and two sliding cephalic screws which are set at a 135 degree angle to allow and facilitate controlled fracture compression. PCCP is indicated for the treatment of trochanteric fractures with intact lateral walls (classified as AO 31.A1 and 31.A2) [18, 19]. The advantages of PCCP include minimal injury to the muscle and tendon structures [13], shorter surgical time, reduced soft tissue damage, reduced need for blood transfusion, and decreased incidence of complications [20].

The purpose of this prospective, multi-center, observational study was to evaluate the functional and clinical outcomes at 1 year after the treatment of trochanteric fractures with the PCCP (Orthofix S.R.L., Verona, Italy) in 8 Italian departments of traumatology.

## Materials and methods

From March 2008 to April 2009, 273 consecutive patients with a stable or unstable proximal femoral fracture, type 31.A1 or 31.A2 according to AO classification, were enrolled in 8 Italian hospitals and followed up until May 2010. Patients with femoral fractures classified as AO 31.A3 multiple concomitant fractures, pathological fractures, active infection in the surgical site, history of previous fractures resolved with a reduced function, or residing outside the geographical region of the treating hospital were excluded from this study.

PCCP was used for the treatment of trochanteric fractures in all the cases. At admission, demographic and clinical data, including age, gender, weight, height, comorbidities (categorized as smoking status, history of alcoholism, diabetes, dementia, asthma, and heart condition), domicile (alone, with relatives, or at an institution), and radiographs, were collected. The requirement of walking aids, the nature of injury (high energy, low energy, or pathological nature), the AO fracture classification, the date of injury, and the date of hospitalization were also recorded. The “ASA physical status classification scale” [21] was used to assess the condition of the patients before surgery. Pre-injury activity and post-surgery functional recovery were evaluated by using the modified Harris Hip Score (mHHS) [22, 23], a hip function questionnaire used to assess patient’s ability in normal daily activities and to provide information about the range-of-movement without a direct evaluation, present only in the original HHS. In literature, there is no widely accepted method for quantifying the importance of change in HHS. In the past, the HHS results have been presented regarding the proportion of patients who achieved previously described clinical end points, identifying four categories by a decrease of 10 % from the total: excellent 90–100, good 80–89, fair 70–79, and poor <70. These categories, unfortunately, are not applicable in our patient set because the pre-surgery hip score is probably often lower than 70, for the advanced age [24]. More recently, Achten et al. [25] used the method of the minimal clinically important difference (MCID) and defined it as a score of 7 points. However, as reported by Smith et al. [26], the MCID for the mHHS is unknown. Therefore, on the basis of the 10 % variation used by Harris, a variation of mHHS ≥ 10 % was arbitrarily assumed to be clinically significant. Patients with a negative variation of mHHS from pre-surgery <10 % were considered well recovered, patients with a variation ≥10 and <20 % fairly recovered, and patients with a variation ≥20 % poorly recovered.

The lowest hemoglobin (Hb) and hematocrit (Ht) levels and the number of blood units transfused during preoperative hospitalization were recorded. Data collected intraoperatively included the delay of surgery, duration of surgery (skin-to-skin time), fluoroscopic time, number of transfusions, and occurrence of any intraoperative complications. During the hospital stay, postoperative assessments consisted of the lowest Hb and Ht values, units of blood transfused, length of skin incisions, pain perceived by the patient in the surgical region 5 days after surgery as assessed by a visual analogue scale (VAS), and occurrence of any complications. Postoperative X-rays were taken. The total length of the hospital stay and the domicile of the patient after discharge were also recorded.

Follow-up visits were scheduled according to the standard practice of the hospitals, namely at 6 weeks (±7 days), 3 months (±30 days), and 1 year (±30 days), and involved functional assessment (mHHS), clinical and radiological assessment, and recording of complications. Radiological assessments, which included anteroposterior (AP) and lateral radiographs of the affected hip, were used to classify the fracture according to the AO classification and to monitor the progress of fracture healing and possible complications.

**Fig. 1:**
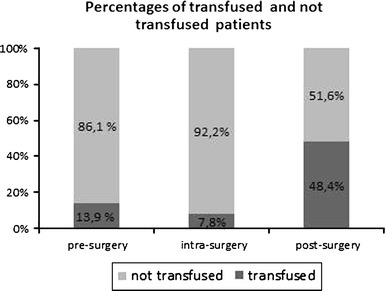
Percentages of transfused and not transfused patients

The primary outcome variable was recovery of the pre-fracture functional status 1 year after the surgery, as previously described. Secondary outcome variables were blood loss, surgical time, complications, and mortality (Fig. 1).

Data were analyzed descriptively. Continuous variables were summarized as mean (SD) and median values, while categorical variables were summarized as counts and percentages. All analyses were performed using SAS version 9.2 (SAS Institute, Cary, NC, USA).

## Results

A total of 273 patients were enrolled in the study. Of these, 208 (76.2 %) were women and 65 (23.8 %) were men. Table 1 shows the baseline characteristics of the patients. ASA score was 3 or 4 in 72.5 % patients; 44 patients (16.1 %) had no comorbidities, 104 patients (38.1 %) had 1 comorbidity, 70 patients (25.6 %) had 2 comorbidities, 42 patients (15.4 %) had 3 comorbidities, 10 patients (3.7 %) had 4 comorbidities, and 3 patients (1.1 %) had 5 comorbidities. The most common (117 patients) concomitant pathology was a cardio-circulatory condition, which included also hypertension. The vast majority of patients (90.8 %) had low energy trauma injuries; all fractures were closed and classified as AO 31.A1 (57.5 %) and 31.A2 (40.7 %), the images were not centrally collected for 5 patients (1,8 %) and the surgeons classified the fractures as AO31.A without specifying if A1 or A2.

**Table 1:** Baseline characteristics of patients

No. of patients	273
Age	
Mean ± SD	82.04 ± 9.93
Median	84
Range	17–104
Gender	
Female	208 (76.2 %)
Male	65 (23.8 %)
Comorbidities	
Cardiac condition	117 (42.9 %)
Central nervous system condition	62 (22.7 %)
Vascular condition	52 (19.3 %)
Other	127 (46.5 %)
ASA classification	
1	6 (2.2 %)
2	69 (25.3 %)
3	173 (63.4 %)
4	25 (9.1 %)
Nature of injury	
High energy	15 (5.5 %)
Low energy	248 (90.8 %)
Missing	10 (3.7 %)
Type of fracture	
Closed	273 (100 %)
Open	0 (0 %)
AO fracture classification	
A1	157 (57.5 %)
A2	111 (40.7 %)
Missing	5 (1.8 %)

At the end of the 1-year follow-up period, 48 patients (17.6 %) had died, and 28 patients (10.2 %) were lost to follow-up. Four patients (1.5 %) were excluded from functional analysis at 1 year for the following reasons: 2 patients did not come to the final follow-up examination, 1 patient could not answer to the HHS questions because of a significant cognitive impairment, and 1 patient was re-operated. Thus, a total of 193 patients (70.3 %) were available for the final evaluation.

**Fig. 2:**
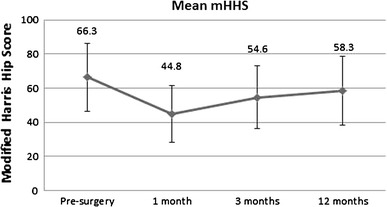
Modified Harris Hip Score pre-surgery and at follow-up visits

Basal and final mHHS values were available only for 181 patients. mHHS values are reported in Fig. 2. At the 1-year follow-up visit, the mean mHHS reduction was 10.2 points. Patients recovered an average 87 % of their pre-trauma score; 94 patients (51.9 %) recovered or improved their pre-fracture mHHS, the score decreased more than 10 % in 30 patients (16.6 %), and more than 20 % in 57 patients (31.5 %). With respect to walking abilities, prior to surgery 107 patients (59.1 %) did not required any aids, 59 patients (32.6 %) used 1 aid, 14 patients (7.7 %) used aids, and 1 patient was unable to walk. At the 1-year follow-up, 49.1 % of patients improved or maintained their walking abilities; 35 patients (19.3 %) walked without aids, 95 patients (52.5 %) used 1 aid, 33 patients (18.2 %) used 2 aids, and 18 patients (9.9 %) were unable to walk. At baseline, 32.2 % of patients lived alone, 59.9 % with relatives, and 7.9 % in a retirement home. At the 1-year follow-up visit, 21.5 % of patients lived alone, 62.7 % with relatives, and 15.8 % in a retirement home. Overall, a low mortality rate was reported. No patients died intraoperatively, 7 patients (2.6 %) died within 30 days, and 20 patients (7.3 %) died within 3 months. At 1-year follow-up period, the overall mortality rate was 17.6 % (48 patients).

Table 2 shows the intraoperative data. The average time between trauma and surgery was 4.6 (2.8) days. The average duration of the fluoroscopic exposure was 44.4 (27.75) s, while the mean duration of surgery was 44.1 (16.55) min. The mean length of the proximal incision was 31 (9.3) mm, while that of the distal incision was 40 (7.2) mm. Intraoperative complications were seen in 6 patients (2.3 %). In 4 patients, the surgeons experienced difficulties in placing the cephalic screws due to narrow femoral necks; in 1 patient the drill tip broke; and in 1 patient, the diaphyseal screw was too short. Following surgery, the patients were discharged after an average duration of 10.7 (4.99) days; the mean total duration of hospitalization was 15.3 (5.96) days. The mean pain score on VAS at day 5 post-surgery was 3.9 (1.38). The mean reduction in Hb levels between the preoperative and postoperative lowest value was −2.8 (1.57) g/dL, while that in Ht levels was −7.9 % (5.74 %). About 13.9 and 7.7 % of patients required blood transfusion pre-surgery and intraoperatively, respectively. Furthermore, 51.3 % of patients did not require transfusions post-surgery, 13.3 % required only 1 unit of blood, 30.6 % required 2 units, and 4.8 % of patients required 3 or more units. Overall, an average of 0.9 (1.09) units of blood was transfused post-surgery.

**Table 2:** Intraoperative data

	*n*	Mean ± SD	Median	Range
Days between admission and surgery	273	4.6 ± 2.8	4	0–16
Fluoroscopy time (s)	250	44.4 ± 27.7	39.5	2–220
Duration of surgery (min)	271	44.1 ± 16.5	41	20–180
Hb (g/dL)				
Pre-surgery	271	12.1 ± 1.6	12.1	7.6–16.5
During hospitalization	265	9.4 ± 1.5	9.3	3.2–14.2
Ht (%)				
Pre-surgery	271	36.3 ± 5.9	36.1	23–94
During hospitalization	265	28.4 ± 4.2	28.1	20–43
Pain at day 5 (VAS)	237	3.9 ± 1.4	4	1–8

Postoperative complications were seen in 12 patients (4,3 %). The major complications included 2 fracture collapses (1 associated with a cut-out and another due to a new fall), 1 case of excessive sliding of the cephalic screw leading to a partial fracture collapse, and 1 case of backing out of the diaphyseal screws. The patient with the backing out of diaphyseal screws was re-operated on; the plate was removed and a hip prosthesis implanted. Minor complications included decubitus ulcers in 3 patients, infections at the surgical incision site in 3 patients, and subcutaneous hematoma in 2 patients. No delayed union or non-union was observed in the group.

## Discussion

In this study, we assessed the functional and clinical outcomes in trochanteric fracture patients treated with PCCP. Our results suggest that treatment with PCCP seems to be associated with good clinical outcomes, low number of blood transfusions, and minimum reduced incidence of intra- and postoperative complications and good functional results in the majority of the patients. These findings are particularly significant considering that 72.5 % of patients in our study had an ASA score of 3 or 4 with several comorbidities. The primary goal of fracture treatment is returning patients to their pre-fracture functional status [4, 5]. The mHHS is considered to be a reliable measure of functional outcome and accounts for pain and functional recovery of the patient, including the daily activities and walking capabilities [22]. In our study, we observed that 51.9 % of patients recovered or improved their pre-fracture mHHS at 12 months post-surgery. In accordance to our results, another study [27] showed a good functional recovery in patients treated with PCCP. In addition, the same study showed also that PCCP conferred better long-term functional recovery than DHS. The improved recovery could be a result of pain reduction and decreased soft trauma associated with the minimally invasive PCCP technique. Mobility is also an important functional outcome to be considered after hip surgery [8]. In a prospective, randomized, controlled clinical trial including patients with intertrochanteric femoral fractures, 18 % of the patients treated with Gamma nail achieved their pre-fracture independent mobility [28]. In comparison, 49.1 % of patients in our study maintained or showed improvement in their walking abilities at the 1-year follow-up visit. In addition, 71.8 % of patients were able to walk without support or with a single aid at the end of 1 year. Laufer et al. [27] reported that patients treated with PCCP ambulated with fewer assistive devices in comparison with those treated with DHS, which suggests that PCCP enhances the functional abilities of patients. A plausible explanation for better mobility could be increased fracture stabilization with the device or lesser damage of pertochanteric and thigh muscles. Change in domicile status of the patients also plays an important role in post-surgical functional recovery. In 1024 patients treated with the sliding hip screw, 83 % returned to their own homes at 1 year, but many required extra care as compared to pre-fracture [29]. Beringer et al. [8] observed that 68 % of patients were residing at home 1 year after fracture treatment. We have to consider that in these studies, patients have lower age or ASA score than our patients. In our study, 66.6 % of patients living alone pre-surgery maintained their domicile and continued living alone, at the 1-year follow-up visit. Functional outcomes in patients treated with PCCP, as evaluated by the improved mHHS, return to pre-fracture mobility, and maintenance of pre-surgery domicile status at the 1-year follow-up period should be considered excellent when compared to other studies and considering the status of our patients.

Studies have reported shorter surgical time with PCCP than with DHS and other devices [17, 25, 30, 31]. In a prospective, randomized study, Janzing et al. [17] reported surgical duration of 65 min with DHS as compared to 49 min with PCCP. Results from a randomized, controlled trial by Peyser et al. [30] showed that the mean operative time in patients treated with PCCP was 67.5 min compared to 82.7 min in patients treated with CHS. A meta-analysis of 3 head-to-head trials comparing PCCP and DHS also reported shorter operative times with PCCP [31]. The average duration of surgery in our study was 44.1 min, which is considerably less than those reported in the above-mentioned studies. A reduced operating time is desirable, especially in elderly patients with comorbid conditions. Fluoroscopy time of over 100 s has been reported in most studies with alternative treatment devices [23, 32]. Saudan et al. [32] reported a fluoroscopy time of 180 s with DHS, and Knobe et al. [23] reported a fluoroscopy time of 143 s with PCCP versus 146 s with DHS and 280 s with the proximal femoral nail (PFN). The radiation time in our study was 44.4 s, which is approximately one-third of the time previously reported and is in line with our shorter operative time.

Previous studies have shown that the PCCP procedure is associated with reduced blood loss and reduced transfusion requirements [13, 30], which leads to faster functional recovery. In the present study, 51.6 % of patients did not require postoperative transfusions. On the contrary, treatment of femoral fractures with the DHS has resulted in higher transfusion rates [31]. The mean decrease in Hb in patients in our study was 2.8 g/dL; this is in agreement with a mean Hb decrease of 3.0 g/dL reported by Peyser et al. [33]. Brandt et al. [20] showed an absolute risk reduction of 45 % for blood transfusion with the use of PCCP. In our study, an average 0.9 units of blood was transfused, which is lower than the 1.2 units with PCCP and 1.7 units with CHS reported in a randomized, prospective trial in patients with intertrochanteric hip fractures by Kosygan et al. [34]. In conclusion, in comparison with other devices, the treatment of trochanteric fractures with PCCP reduced the rates of postoperative transfusions, thanks to the fact that the plate is inserted percutaneously and there is a minimal blunt dissection of the muscles.

Postoperative pain impedes ambulation, increases patient discomfort, and hence delays recovery. In the present study, the pain score on VAS at 5 days post-surgery was 3.9 (1.38). This result is in agreement with other studies in which it was demonstrated that patients treated with the PCCP experienced lower pain scores on VAS compared to patients treated with CHS [27]. Thus, the PCCP appears to be well tolerated by patients, probably due to less damage to muscles. The mean duration of hospitalization was reported to be 37 days and 17 days with the Gamma nail [35] and DHS [36], respectively. The average duration of hospitalization was 15 days in our study. However, we recognize that duration of hospitalization can be influenced by organizational matters. In some cases, the discharge was postponed until an appropriate nursing home could be found to begin rehabilitation of the patient. Short duration of hospitalization in our study could be attributed to the minimally invasive PCCP technique, which leads to reduced tissue trauma, faster recovery, and early discharge.

About 90 % patients did not experience postoperative complications in our study. Our findings are in agreement with a retrospective study by Yang et al. [37], in which 82 % of patients did not have postoperative complications. Brandt et al. [20] also reported less complications with PCCP than with DHS. A trend toward decreased incidence of postoperative infections with the PCCP was also observed in a meta-analysis by Panesar et al. [31].

Overall, the mortality rate in our study was 17.6 %. Similar values were observed by Bensafi et al. [13]. Mortality rates ranging from 18 to 33 % have been previously reported in studies with hip fractures at the end of 1 year [37, 38]. In a meta-analysis comparing PCCP and DHS, Panesar et al. [31] reported a decrease in overall mortality with PCCP. A mortality rate of 11.4 % at 3 months post-surgery was reported by Berkenbaum et al. [39]. In comparison, our study was associated with a mortality rate of 7.33 % at 3 months post-surgery. The low mortality rate in our study can be attributed to reduced blood loss, surgical trauma, postoperative complications, and accelerated recovery. These results are particularly important, since 72.5 % patients in our study had an ASA score of 3 or 4 with a high number of comorbidities. The ASA rating is a good predictor of mortality [40–42]. Patients aged 65–84 years with an ASA score of 3 or 4 have a poorer health and have a higher mortality rate as compared to patients who are classified as ASA 1 or 2, regardless of hip fracture. Hip fracture may contribute to this increased mortality, as frail patients are less likely to survive the insult of a major fracture and surgery [42]. However, with the mini-invasive PCCP approach, elderly patients with associated comorbidities are less exposed to the hazards of blood transfusion, such as hemodynamic compromise and potential infections [30]. Thus, PCCP could be the treatment of choice in highly compromised patients with associated comorbidities. Paksima et al. [43] reported that patients with high ASA score of class 3 or 4 were at an increased mortality risk following hip fractures. The mortality rate at 1 year was reported to be nine times higher in patients with ASA score 3 and 4 than in healthy or mildly affected patients (ASA scores 1 and 2) [44]. In the present study, in spite of higher proportion of patients with ASA scores 3 and 4, better functional outcomes and lower mortality rates were observed when compared to the other studies.

The following study limitations have to be considered while interpreting our results. This was an observational study; therefore, surgeons followed the standard care without additional visits or examinations. There was no control group included in our study, so we have compared our results with other published studies. The study was conducted across 8 different hospitals, which may cause variations in the standard of care during hospitalization and the follow-up period, resulting in heterogeneity of data. When the radiographs were not available at the end of 1-year follow-up, the case report form was compiled with a telephonic interview.

In our study, it has been demonstrated that the treatment of trochanteric fractures with PCCP is associated with good recovery of clinical and functional outcomes in terms of the mHHS, mobility, and domicile status of the patient. PCCP also offers additional advantages such as reduced surgical time, blood loss, postoperative complications, and mortality rate as previously reported.
